# Diminished Behavioral and Neural Sensitivity to Sound Modulation Is Associated with Moderate Developmental Hearing Loss

**DOI:** 10.1371/journal.pone.0041514

**Published:** 2012-07-26

**Authors:** Merri J. Rosen, Emma C. Sarro, Jack B. Kelly, Dan H. Sanes

**Affiliations:** 1 Department of Anatomy and Neurobiology, Northeast Ohio Medical University, Rootstown, Ohio, United States of America; 2 Center for Neural Science, New York University, New York, New York, United States of America; 3 Department of Biology, New York University, New York, New York, United States of America; 4 Department of Psychology, Carleton University, Ottawa, Ontario, Canada; University of Auckland, New Zealand

## Abstract

The acoustic rearing environment can alter central auditory coding properties, yet altered neural coding is seldom linked with specific deficits to adult perceptual skills. To test whether developmental hearing loss resulted in comparable changes to perception and sensory coding, we examined behavioral and neural detection thresholds for sinusoidally amplitude modulated (sAM) stimuli. Behavioral sAM detection thresholds for slow (5 Hz) modulations were significantly worse for animals reared with bilateral conductive hearing loss (CHL), as compared to controls. This difference could not be attributed to hearing thresholds, proficiency at the task, or proxies for attention. Detection thresholds across the groups did not differ for fast (100 Hz) modulations, a result paralleling that seen in humans. Neural responses to sAM stimuli were recorded in single auditory cortex neurons from separate groups of awake animals. Neurometric analyses indicated equivalent thresholds for the most sensitive neurons, but a significantly poorer detection threshold for slow modulations across the population of CHL neurons as compared to controls. The magnitude of the neural deficit matched that of the behavioral differences, suggesting that a reduction of sensory information can account for limitations to perceptual skills.

## Introduction

The loss of sensory experience leads to profound effects on the form and function of central neurons, and these changes are thought to disrupt sensory perception in adulthood. Support for this theory draws largely from studies in which developing animals are unilaterally deprived of sensory stimulation. In the visual system, studies of amblyopia demonstrate a compelling correlation between cortical encoding mechanisms and deficits in visual perception [1]. Similarly, in the auditory system, unilateral hearing loss leads to changes in binaural coding properties that are closely associated with perceptual deficits [2], [3], [4], [5], [6], [7]. The deleterious effects of unilateral deprivation are thought to reflect the competition between active and inactive pathways. However, even the original studies of bilateral visual deprivation noted that 79% of primary cortical cells responded abnormally or were unresponsive to sensory stimulation [8]. Moreover, moderate binaural deprivation during development has a broad impact on synaptic function in auditory cortex [9], [10], [11], [12], [13], and these changes persist in adults [14]. Taken together, these findings suggest that developmental auditory deprivation should lead to deficits in auditory detection tasks, with clear correlates to neural encoding, even in the absence of neural competition. Here, we tested this idea by measuring behavioral and neural detection thresholds for amplitude modulated stimuli in the gerbil.

Animal communication sounds are composed of low frequency amplitude fluctuations and, for humans, these envelope cues are necessary and sufficient for speech comprehension [15], [16], [17], [18], [19]. The detection of small fluctuations, commonly assessed with sinusoidally amplitude modulated (sAM) tones or noise, displays a relatively prolonged maturation [20], [21], [22] and may remain vulnerable to developmental deprivation. Human studies suggest that early hearing loss perturbs temporal processing [23], [24]. Furthermore, children with cochlear implants have poorer sAM sensitivity than implanted adults, suggesting a greater effect of early auditory deprivation [25].

Prolonged conductive hearing loss (CHL), which can occur as a consequence of chronic otitis media, has been associated with deficits in perception and speech processing [26]. However, it is challenging to establish the long-term effects of developmental hearing loss in humans because most studies consider individuals with different ages of hearing loss onset, and who receive varying levels of remediation. Furthermore, most developmental studies consider subjects with significant inner ear damage (e.g., sensorineural hearing loss), such that deficits in temporal processing may reflect a reduction of cochlear compressive non-linearities [27], as well as central factors. To circumvent these problems, we induced CHL in gerbils just prior to hearing onset [28] and measured behavioral and neural sAM detection thresholds in adulthood, letting us assess nervous system function independent of cochlear function.

## Methods

### Animals

All procedures relating to the maintenance and use of animals were in accordance with the “Institutional Animal Care & Use Committee Handbook” and were approved by the University Animal Welfare Committee at NYU. Male and female gerbil pups (*Meriones unguiculatus*) were weaned from commercial breeding pairs (Charles River) at postnatal day (P) 30 and maintained in a 12-h light/12-h dark cycle. Behavioral data were obtained from 24 adult control (CTR) animals (P65–125, 9 males and 15 females) and 23 animals with developmental CHL (P65–125, 15 males and 8 females). The groups were comprised of animals from multiple litters. All animals that entered the behavioral protocol were included in the analyses. No selection criteria were imposed, as is common in behavioral studies. Therefore, poor performers were not eliminated during any phase of the procedure, allowing us to compare both mean performance and between-animal variability within and across treatment groups. Physiological data were obtained from separate groups of adult CTR and developmental CHL gerbils: 15 adult CTR (P60–81, 9 males and 6 females) and 8 CHL (P61–90, 5 males and 3 females).

### Behavioral Training and Testing

#### Experimental environment

Behavioral data were collected as previously described [22]. Gerbils were placed in a small wire cage in a room lined with echo-attenuating material, and observed in a separate room via a closed circuit monitor. The test cage contained a stainless steel drinking spout and metal floor plate. When the animal contacted both the plate and spout, a circuit was completed that initiated water delivery via a syringe pump (Yale Apparatus). A personal computer, connected to a digital I/O interface (Tucker-Davis Technologies) measured animal contact and controlled the timing of acoustic stimuli, water delivery (0.25 ml/min), and a small current delivered at the end of warning trials. Auditory stimuli were generated by the Tucker-Davis Technologies system and delivered via a calibrated tweeter (KEF Electronics) centered 1 m in front of the test cage, with the speaker ∼20 degrees above the animal’s head. Sound level at the test cage was measured with a spectrum analyzer (Brüel & Kjaer 3550) via a 1/4 inch free-field condenser microphone positioned at the head location when in contact with the spout. Noise levels are in units of dB SPL, converted from RMS measurements.

#### Auditory stimuli

CTR and CHL groups were divided into subgroups that were trained on sAM modulation frequencies (MF) of either 5 Hz or 100 Hz. Separate subgroups were used for the two MFs to ensure that all subgroups had equivalent experience with their tasks. The stimulus was broadband noise with a low frequency falloff of 25 dB at 3.5 kHz and a high frequency falloff of 25 dB at 20 kHz. The sound pressure level remained constant during both the pre-trial and warning intervals to exclude the use of an energy cue. Each trial was 2500 ms total length, comprised of a pretrial interval containing 1200 ms of unmodulated noise, 1000 ms of either a modulated noise (warn trial) or a continuation of the unmodulated noise (safe trial), and 300 ms of unmodulated noise. The spout was monitored for contact during the final 500 ms of the pretrial interval, and the trial proceeded only if the animal remained in contact with the spout for >50% of this interval. The warning stimulus was 1000 ms of sinusoidally amplitude modulated noise at a modulation frequency of either 5 or 100 Hz (depending on experimental group) and at varying depths. Warning stimuli were followed immediately by an aversive unconditioned stimulus (300 ms electrical current delivered via the lick spout). To determine whether the animal detected the warning stimulus, contact with the spout was monitored during the final 100 ms of the warning stimulus. During this window, a contact time of <50 ms was scored as a hit. For the same window during safe trials (where the entire 2500 ms duration consisted of unmodulated noise), a contact time of <50 ms was scored as a false alarm (FA). Warning trials always occurred at the end of a block of 2–4 safe trials, randomized to avoid temporal conditioning.

#### Sound levels

In order to choose appropriate amplitude levels for the two experimental groups, task-specific sound level thresholds for a 100% MD sAM were assessed in pilot groups of CTR (n = 6) and CHL (n = 6) animals. Trained animals were tested with 100% MD sAM noise at varying amplitudes using a staircase procedure; levels were moved up and down in 3 dB increments based on performance, and threshold was determined as the level where detection dropped below 50%. Experimental levels were then set to be ∼20 dB above task-specific threshold: CTR animals were presented signals at 35 dB SPL, and CHL animals were presented signals at 70 dB SPL. For the experimental animals, it was not possible to obtain task-specific level thresholds from each animal prior to sAM detection testing for two reasons: 1) animals needed to be very practiced to yield accurate level thresholds and 2) presenting difficult stimuli (e.g., low sound level) early in training can extinguish the learned behavior. Thus individual experimental animals had their task-specific thresholds measured after completion of all stages of training and testing.

#### Procedural training

All training used a conditioned avoidance procedure [29], [30]. Animals were first placed on controlled water access, and upon introduction to the experimental cage, learned to obtain water from a lick spout in the presence of an unmodulated noise stimulus, while contact with the waterspout was monitored. Animals were then trained to withdraw from the spout when an acoustic cue (modulation of amplitude) was present. To train the withdrawal response, a low AC current (0.5–1.0 mA, 300 ms; Lafayette Instruments) was delivered through the waterspout immediately after the warning signal. Since animals display large between-subject variability in pain sensitivity [31], [32], [33], the shock level was adjusted continuously for each animal to reliably produce withdrawal from the spout without dissuading an animal from approaching the spout on subsequent trials. To establish criterion performance on the procedure, warning trials (100% modulation depth) were presented until performance reached a criterion of ∼70% correct over 10 consecutive trials.

#### Assessment of sAM detection thresholds

Once animals in each experimental group reached criterion on the conditioned avoidance procedure, we obtained an initial assessment of sAM detection threshold by testing animals on a broad range of sAM modulation depths (MD; 10–100% MD in 10% steps) presented ten times each in a randomized order, with the same order being delivered to each animal. In order to obtain minimum thresholds, animals underwent 7–10 days of additional testing. Each day, a small range of MDs (five in total, divided evenly into steps of 10% depth) was repeatedly presented in descending order, bracketing each animal’s initial detection threshold. On each subsequent day, an animal’s performance on the previous day determined the range of MDs on which it was tested (i.e., always bracketing the previous detection threshold). As animals were challenged with harder values based on their performance, thresholds across training days are an indicator of improvement. Thresholds obtained during the final three days and the best day were taken as a measure of practiced detection.

#### Behavioral data analysis

A performance value, d′ = z(false alarm) - z(hit), was obtained for z scores that corresponded to the right-tail p values [34], [35], and was calculated for each MD. Thresholds were defined as the sAM depth at which performance reached a d′ = 1 and only sessions in which animal performed a minimum of 25 trials were included in the analysis. Psychometric functions of d′ across MD were constructed from the initial assessment with random stimuli and throughout each day of additional testing. The best or average performance during the final 3 days of testing served as the assessment of practiced sAM detection thresholds. Performance during training to criterion (when animals received warning trials of 100% MD) was quantified across all training trials (excluding the first 10) as average #hits - average #FAs. All values are given as mean ± standard error (SEM).

### Neurophysiology

Some of the control neurons presented here were used in a previous study [36]. That work included neurons with a range of best modulation frequencies (BMF; defined below). This study analyzes the subset of those cells with BMFs of 5 Hz, to match the behaviorally-tested MF (with the exception of responses across multiple modulation frequencies and for static tones).

#### Surgical preparation and chronic recordings

Gerbils were premedicated with ketoprophen (1.5 mg/kg, i.n.) and dexamethasone (0.35 mg/kg, i.p.) and hydrated with normosol (1.5 ml, s.c.). The animals were anesthetized with isoflurane and held in a stereotaxic apparatus. A small headpost was positioned along the midline and secured with dental acrylic, and a silver ground wire was implanted into the posterior contralateral skull. A craniotomy was made over the left temporal cortex caudal to the bregma suture using stereotaxic coordinates [37], and the dura was left intact. A thin well of dental acrylic was built along the perimeter of the craniotomy, the cortical surface was covered with silicone oil, and the craniotomized area was covered with a disposable cap of silicone elastomer (Sammons Preston Rolyan, Bolingbrook, IL).

Animals were placed in a soundproof chamber (Industrial Acoustics Company, Bronx, NY), and the head was stabilized using the headpost. During chronic recording sessions, animals stood comfortably on a platform and were free to move their limbs while the head position remained fixed. If an animal exhibited heightened anxiety or restlessness, medetomidine (a relaxant that acts on 2-adrenoceptors) was administered intranasally (0.1–0.3 mg/kg). Gerbils trained to detect amplitude modulation are able to perform the task after receiving similar doses of medetomidine (D. H. Sanes, unpublished observations), and medetomidine does not produce an observable effect on neuronal activity [38]. Thus the results for cells recorded with and without medetomidine are presented together.

The silicone elastomer cap was removed at the beginning of each session and a fresh cap applied at the end. The dura was covered with saline during recording to maintain moisture. Platinum-plated tungsten electrodes (1.5–2.5 MΩ; MicroProbe, Gaithersburg, MD) were advanced ventrally through the craniotomy with an electrode tip angle of 14° lateral to vertical to isolate neurons in primary ACx. Single-unit recording procedures were identical to those described previously [36].

#### Acoustic stimulation for neurophysiology

The system used for stimulus generation and sound delivery (MALab, Kaiser Instruments) as well as the calibration procedure have been described previously [39]. Calibrated acoustic stimuli were presented to each ear through an electrostatic speaker coupled to a custom-made ear insert. All stimuli were presented monaurally to the right ear except in cases in which a monaural stimulus failed to elicit reliable responses, in which case a binaural stimulus was used.

To assess responses to static tones, neurons were presented with tone pips (200 ms, 5 ms cosine-ramped rise/fall time). First, the frequency range over which the neuron was responsive was obtained with an iso-intensity function at 10–30 dB above threshold (threshold criteria are described below). This was followed by a rate-level function (RLF) at the BF of the unit, measured at increments of 5 dB SPL, for 10–20 trials (with a 1 sec intertrial interval).

To assess responses to sAM, each cell was presented with sAM tones (2 s duration with 10 ms rise/fall) at BF, at the SPL that produced the strongest response. This was typically 40 dB above the cell’s threshold. Modulation transfer functions (MTFs) were obtained by presenting five or ten trials of multiple modulation frequencies (1, 2, 5, 10, 20, 50, and 100 Hz) at 100% modulation depth with an intertrial interval of 1 sec. An unmodulated tone at the same frequency and SPL and of the same duration as the SAM tones was included in the stimulus sequence as a control. The best modulation frequency (BMF) was determined from the MTF as the modulation frequency eliciting the highest response strength (i.e., the product of firing rate and vector strength). Modulation depth functions (MDFs) were then obtained by presenting ten trials of multiple modulation depths at BF and BMF with an intertrial interval of 1 sec. The following modulation depths were typically presented to each ACx neuron: 5, 10, 20, 50, 70, 80 and 100% MD.

#### Neurophysiological data analysis: RLFs and sAM responses

From RLFS, mean first-spike latency over 10 or more trials was calculated at BF and the SPL that produced the maximum response. Firing rates to tones were calculated over a time window equal to the stimulus duration. Maximum firing rates were calculated at the sound level that elicited the highest firing rate. Threshold, dynamic range and monotonicity were determined from the RLF. Threshold was defined as the dB SPL level below which there was at least a 20% increase in firing rate, stepping up from one dB SPL level to the next; threshold firing rates were calculated at this sound level. Dynamic range was defined as the range between the dB SPL levels where each cell responded at 10% and 90% of its maximum firing rate, calculated by interpolation. To allow valid comparisons, RLF data were collected using equivalent ranges of levels across the two groups (0 to 90 dB SPL for CTRs, 30 to 120 dB SPL for CHLs). Group differences for each of these were assessed with t-tests. Non-monotonic neurons were defined as those whose firing rates at the highest dB SPL tested dropped below 50% of their maximum firing rate. Group differences for monotonicity were assessed using chi-square.

Responses to sAM tones were analyzed using three measures: phase-locking to the modulation period (vector strength), firing rate, and power at the modulation frequency (referred to hereafter simply as power). These three measures were used to construct MTFs and MDFs based on phase locking, firing rate, and power. The first modulation period was excluded to eliminate contamination by the onset response [40]. First, vector strength [41] was calculated by treating every spike as a unit vector in a polar coordinate system, where the angle was the instantaneous phase, and the mean phase of the response was the direction of the sum of those vectors, normalized to the firing rate. Vector strength (the length of that mean vector) depends on how tightly the spikes cluster about that one point in the stimulus cycle, varying between zero (e.g., a flat period histogram) and one (all spikes at one phase), and is thus a measure of tight phase-locking. Next, the average firing rate was determined for each modulation frequency and modulation depth. The third measure, power, accounts for both temporal and rate information, but does not depend on phase-locked responses, and is thus an indicator of how well the spike pattern matches the shape of the sAM envelope. Power was measured by a discrete Fourier transform of spike times using a multitaper process (CHRONUX Toolbox, Cold Spring Harbor). This analysis provides the magnitude of the spiking response at the modulation frequency of the sAM stimulus [42], measured as power (spikes/sec^2^/Hz). For each measure, group differences for MTFs or MDFs were assessed with ANCOVAs.

#### Neurophysiological data analysis: neurometrics

Two neurometrics were applied to those cells whose best modulation frequency was 5 Hz, in order to predict behavioral detection based on the activity of those cells. First, we converted individual neural depth detection into a measure directly comparable with the behavioral data: d′ curves for each cell were calculated based on power at the 5 Hz modulation frequency of the sAM stimuli. Mean z-scores across trials were calculated for each cell at each depth level. For every depth level, d′ was then calculated as 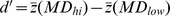, where MD_low_ is the lowest depth tested (5%). Therefore, this is a within-cell neurometric, in that the detection ability of each neuron was compared with its own baseline. For each neuron, threshold for detection of the signal was defined as d′ = 1, the same criteria used for the behavioral analysis.

Second, a pooling neurometric was applied that assumes a neural architecture of a single downstream neuron that receives input from all cells [43], [44]. To measure detection, activity elicited by a baseline stimulus was compared with that elicited by each depth, and the event with maximum likelihood was chosen by a winner-takes-all strategy. Unlike the d′ neurometric, this is an across-cell neurometric, as each neuron did not serve as its own baseline. The neurometric was applied based on power at the 5 Hz modulation frequency of the sAM stimuli. For each experimental group, cells were chosen with replacement from the entire pool of neurons. Rather than sampling the same observed responses repeatedly on each trial, we used the Fano factor ρ as a measure of neuronal variability to simulate responses drawn from distributions whose mean and variance matched those of the empirical measurements. Thus, for each MD and for baseline (5% MD), the mean response across trials for each cell was jittered using 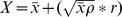, where 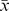 is the mean response magnitude, ρ is the Fano factor, and r is a small random number with mean zero and standard deviation of one. Jittered responses were summed, the summed response magnitudes for baseline versus each MD were compared, and a larger value for the higher MD was considered correct detection. This was repeated 20 times (drawing a random subset for each trial), with each comparison equivalent to a single trial, and performance was measured as average correct detection across trials. The neurometric was run 100 times and depicted as mean ± standard deviation, as its performance was quite variable when using only 20 trials (a value chosen to reflect the collected neural data).

### Surgery for Conductive Hearing Loss

Bilateral conductive hearing loss was induced prior to hearing onset. Gerbil pups at P10–11 were anesthetized with the halogenated ethyl methyl ether methoxyflurane (Metofane). Conductive hearing loss was induced by tympanic membrane puncture and malleus extirpation [45]. A postauricular skin incision was made, the tympanic membrane was visualized and punctured with forceps, and the malleus was removed through this opening. The postauricular wound was closed with cyanoacrylate glue, and the procedure repeated on the other side. At the end of the study, CHL animals were sacrificed and their ears were examined. Malleus removal did not disrupt the stapes in its attachment to the oval window in any of the animals used for this study.

### Auditory Brainstem Responses (ABRs)

After behavioral testing and acquisition of behavioral hearing thresholds, ABRs were measured from a subset of tested animals to measure neural hearing thresholds. Animals were anesthetized with ketamine and chloral hydrate and presented with auditory stimuli as described for neurophysiological recordings. Responses to individual stimuli were conducted using stainless steel needle electrodes inserted subdermally at the dorsal midline between the eyes (non inverting), posterior to the right pinna (inverting), and base of the tail (common ground), and were recorded with a Grass P15 amplifier. Auditory stimuli were 100 µs clicks or 4 ms pure tones with 1 ms rise/fall times, repeated at 11.3/s [45]. Sound level was adjusted in 5 dB steps to obtain a threshold response (i.e., a visually detectable N1 potential).

## Results

### Effect of Developmental Conductive Hearing Loss on Behavioral sAM Detection Thresholds

Behavioral sAM detection thresholds were obtained from two groups of adult gerbils: animals reared with conductive hearing loss (CHL) induced prior to hearing onset, and age-matched controls (CTR). Animals were trained to detect a 100% sAM depth signal (5 Hz or 100 Hz modulation, noise carrier) in a continuous broadband noise.

**Figure 1:**
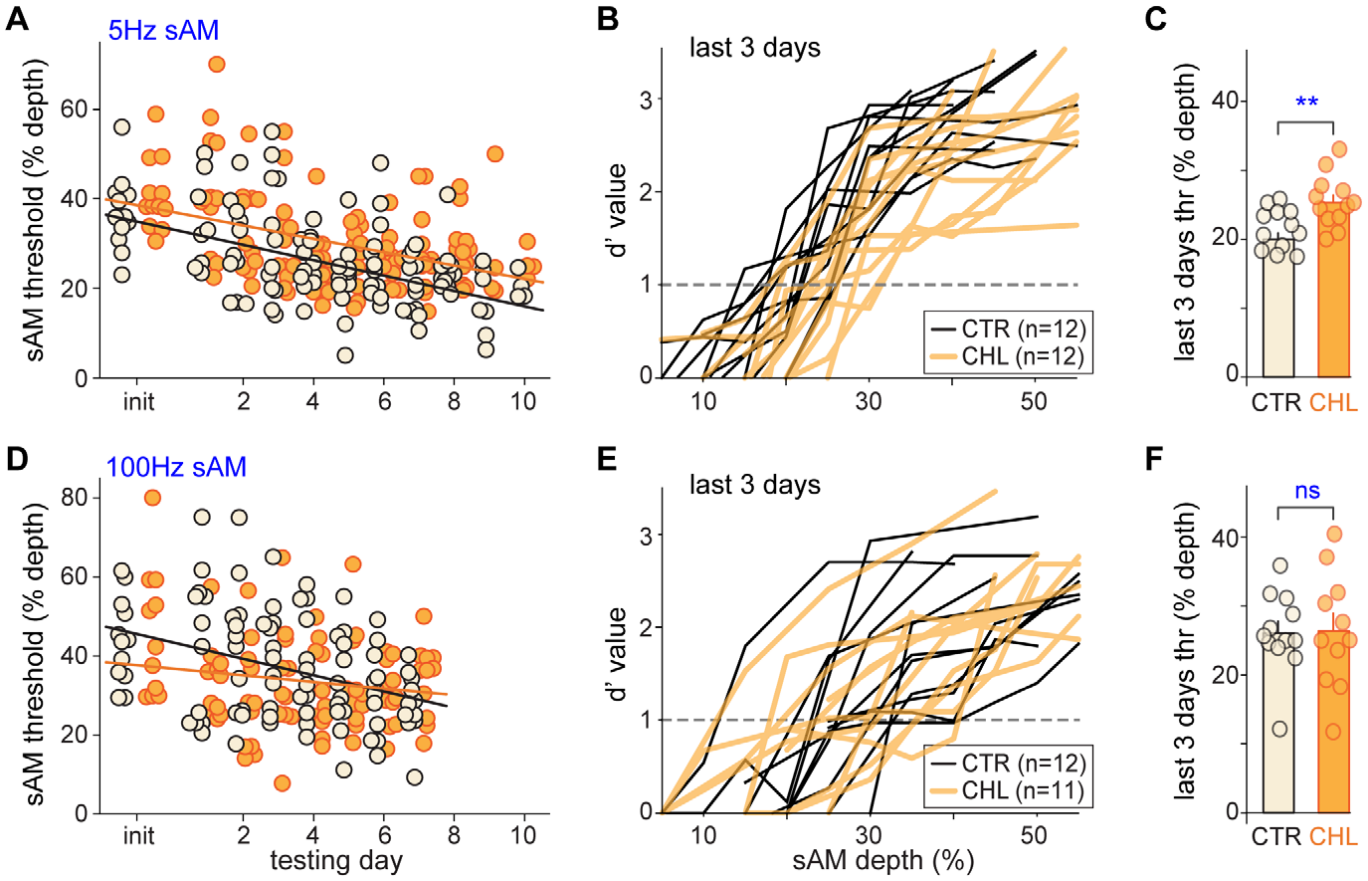
Developmental CHL increases behavioral detection thresholds for slow but not fast sAM. (A) Detection thresholds during initial testing and for all subsequent testing days indicate gradual improvement (linear fitted *lines*) which did not differ across groups. (B) Behavioral detection curves for 5 Hz noise were averaged over the last 3 days of testing. (*C*) Thresholds derived from these curves showed that CHL animals had a small but highly significant increase in detection thresholds. (D) Improvement for 100 Hz sAM detection across testing days (linear fitted *lines*) did not differ across groups. (E) In contrast with slow sAM detection, behavioral curves for 100 Hz sAM detection overlapped in CTR and CHL animals, and (F) depth detection thresholds did not differ. *Black*  =  CTR; *Orange*  =  CHL. **  =  p<.01.

We obtained an initial assessment of detection threshold immediately after each animal reached criterion performance on the task. Animals were tested with a shuffled set of modulation depths (MD) across a broad range (10–100% MD). Detection threshold was defined as the modulation depth at which an animal’s sensitivity was *d′ = 1* (Fig. 1A, *“init”*). To obtain the best detection thresholds, animals were tested over 7–10 days at successively smaller MDs (see Methods). Thus in contrast to the initial testing that spanned a broad range of MDs, each session consisted of a narrow range of MDs that was based on the animal’s performance on the previous session. Thresholds across initial testing and successive testing days (Fig. 1A) show a gradual improvement in performance that did not differ across groups (linear fits for CTR: R = −0.50, p<.0001 and CHL: R = −0.42, p<.0001; ANCOVA interaction measuring differences in slopes of fitted lines: F(8,8) = 0.9, p = 0.53). For the modulation frequency of 5 Hz, CTR animals achieved significantly lower thresholds than CHL animals. Figure 1B shows psychometric functions for each animal during the final three days of testing. The individual thresholds and mean values for the group are in Figure 1C. Thresholds for CHL animals were shifted to higher MDs (CTR: 21.6±0.8%MD vs CHL: 25.6±1.1%MD; t(22) = 2.9, p = .009). A performance difference was also observed when comparing each animal’s best performance day (CTR: 17.2±1.2%MD vs CHL: 21.1±0.9%MD; t(22) = 2.6, p = 0.016). While the range of thresholds overlapped for the two groups, the distribution of CHL thresholds was clearly shifted towards a larger depth. Thus, bilateral developmental CHL impaired detection thresholds for 5 Hz modulated noise.

To determine whether CHL animals are equivalently impaired in detecting higher MFs, we used an identical procedure to test CTR and CHL groups that were trained with 100 Hz sAM. Animals in the two groups showed equivalent improvement in performance over initial and subsequent testing days (Fig. 1D; linear fits for CTR: R = −0.38, p<.0001 and CHL: R = −0.18, p = 0.08; ANCOVA interaction measuring differences in slopes of fitted lines: F(7,7) = 1.3, p = 0.25). Yet in contrast to trained performance on 5 Hz sAM, CTR and CHL animals did not differ when trained and tested on 100 Hz sAM. As shown in Figure 1E, psychometric functions compiled over the last three days of each animal’s performance overlapped for the two groups, as did detection thresholds (Fig. 1F; CTR: 31.6±2.6%MD vs CHL: 27.4±1.9%MD; t(21) = 1.32, p = .20). The same equivalent performance held when comparing each animal’s best performance day (CTR: 24.9±1.7%MD vs CHL: 25.2±2.0%MD; t(21) = 0.1, p = .91). Therefore early-onset CHL did not impair detection of 100 Hz modulations.

### Sensation Level Alone does not Account for the Effect of CHL

**Figure 2:**
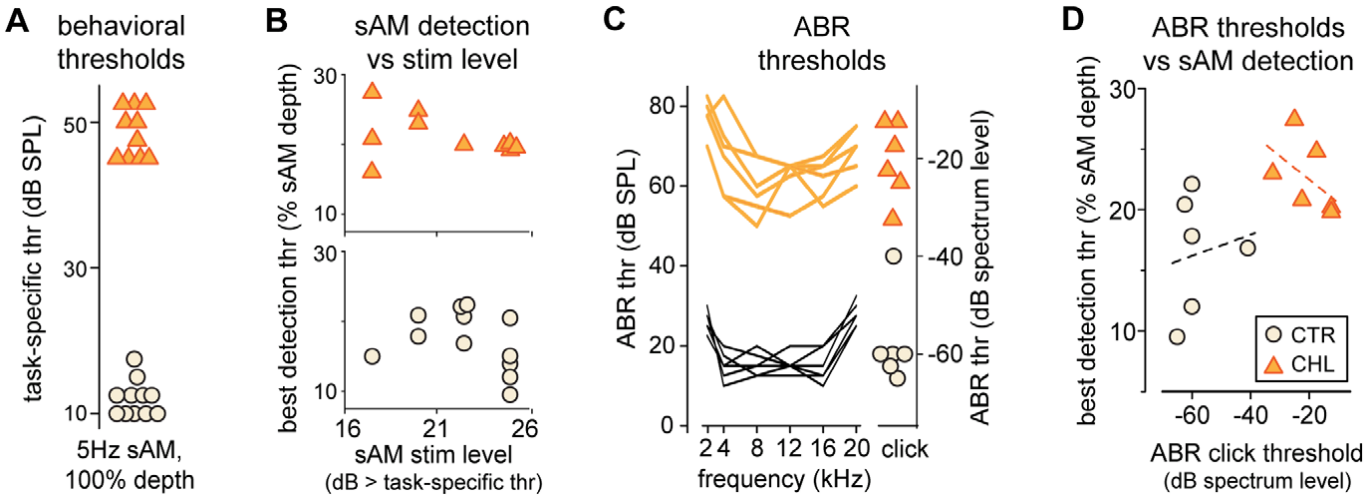
Differences in 5 Hz sAM detection thresholds are not attributable to hearing level, measured behaviorally or neurally. (A) Task-specific behavioral thresholds (the sound level required for detection of a fully-modulated sAM) were predictably increased in CHL animals, by ∼35 dB, indicating the effectiveness of the CHL manipulation. (B) The sAM stimulus level for each animal was the amount above task-specific threshold at which its behavior was tested. In neither CTRs nor CHLs was there a correlation between this stimulus level and sAM detection threshold, indicating that performance differences are not attributable to stimulus level differences. (C) Neural ABR thresholds measured across frequency (*left*) and for clicks (*right*) were predictably increased in CHL animals by ∼40 dB. (D) Consistent with the lack of correlation with behavioral hearing thresholds, in neither CTRs nor CHLs was there a correlation between neural hearing thresholds and sAM detection thresholds. *Black*  =  CTR; *Orange*  =  CHL.

The increased detection thresholds for 5 Hz sAM (Fig. 1C) could be attributable to factors other than developmental experience. One possibility is that stimuli were not delivered at equivalent sensation levels. The two groups were tested with stimuli that differed by 35 dB (35 vs 70 dB SPL for CTR vs CHL, respectively). These sound levels were based on pilot experiments in which sAM task-specific thresholds were obtained (see Methods). It was not possible to obtain task-specific thresholds from each animal prior to sAM detection testing for two reasons: 1) animals needed to be very practiced to yield accurate level thresholds and 2) presenting difficult stimuli (e.g., low sound level) early in training can extinguish the learned behavior. Thus, it was only after obtaining sAM detection thresholds, that we determined each animal’s task-specific sound level threshold for detection of a 100% MD signal. The between group difference for task-specific thresholds (Fig. 2A; CTR: 12.0±0.7, n = 11 vs CHL: 48.5±1.1 dB SPL, n = 10, t(19) = 28.5 p<.0001; three animals stopped performing reliably prior to acquiring level thresholds) was consistent with the levels chosen for assessment of sAM detection. These measurements revealed that individual animals were tested from 16 to 26 dB above their task-specific threshold (Fig. 2B, *x-axis*). However, the stimulus level relative to threshold did not predict sAM detection ability (Fig. 2B). There was no significant correlation between stimulus level (relative to hearing threshold) and best sAM detection threshold for either CTR (R^2^ = 0.05 p = .53) or CHL animals (R^2^ = 0.08, p = .45).

It is also essential to demonstrate that even the quietest portion of the modulated signal (the trough) was fully audible to both groups. Although we did not collect absolute threshold detection levels for the noise carrier, they could not be higher than the task-specific threshold, so we use these values here as an audibility indicator. Across the range of sAM thresholds for each group, the trough dB SPL ranged from 32 to 34 dB SPL in CTRs and 66 to 68 dB SPL in CHLs, both well above the task-specific thresholds shown in Figure 2A. Furthermore, the difference for each animal between trough level at sAM best threshold and task-specific threshold did not differ across the groups (CTR: 21.3±0.9, n = 11 vs CHL: 19.3±1.0 dB, n = 10, t(19) = 1.4 p = 0.17), indicating that the audibility of the trough did not account for the group sAM threshold difference. Therefore, behavioral hearing thresholds did not account for poorer sAM detection in CHL animals.

Another measure of hearing threshold is the auditory brainstem response (ABR). After behavioral testing with 5 Hz sAM was completed, ABRs were measured for a subset of CTR and CHL animals (Figure 2C). In response to clicks, ABR thresholds were 37.5 dB higher in CHL vs CTR animals; averaged across a range of tone frequencies, ABR thresholds were 47 dB higher. However, as was the case for the behaviorally-measured hearing thresholds, these did not predict sAM detection ability. Figure 2D illustrates the lack of correlation between ABR click threshold and best sAM detection threshold for CTR (R^2^ = 0.02 p = .78) and CHL animals (R^2^ = 0.24, p = .32). Thus ABR-based hearing thresholds did not account for poorer sAM detection in CHL animals.

Since we acquired both behavioral and ABR-based hearing thresholds in a subset of animals, we were able to directly compare these measures. Of note is that within either group, there was no significant correlation between behavioral and ABR measures of threshold (CTR: R^2^ = 0.005 p = .90, CHL: R^2^ = 0.008 p = .86).

### Training and Performance Variables do not Account for the Effect of CHL

**Figure 3:**
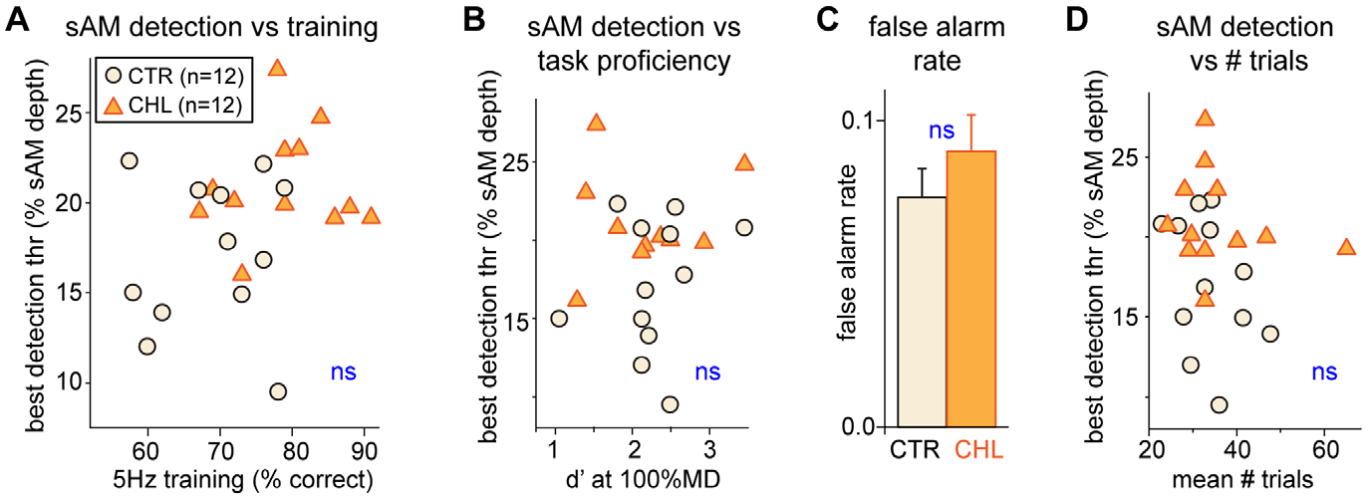
Differences in 5 Hz sAM detection thresholds are not attributable to measures of training or attention. (A) Higher sAM detection thresholds in CHLs were not due to better training, as CHLs reached slightly better compentence during training than CTRs, and criterion performance for detection of a fully-modulated sAM did not correlate with sAM detection thresholds for either group. (B) Proficiency with the task during initial testing, measured by d′ levels for fully-modulated sAM, did not explain differences in sAM detection threshold. (C) False alarm rates, a measure of attention, were not different across groups. (D) The number of trials per session, a measure of motivation, also did not explain differences in sAM detection threshold. *Black*  =  CTR; *Orange*  =  CHL.

The amount of practice or cognitive factors (e.g., motivation, attention) could have contributed to differences in sAM detection threshold. To determine whether CTR and CHL animals were equally proficient at the task, we compared sensitivity for the largest MD (100%) at two time points: during initial testing and over the course of training. Figure 3A (*x-axis*) shows that during the final day of training, CHL animals actually achieved better competence on the basic task (training performance for CTR animals: 69.5±0.09% vs CHL animals: 79.0±0.07%; t(22) = 3.3, p = .003). Furthermore, during the initial testing, d′ detection levels for 100%MD did not differ between groups (CTR: 2.3±0.2 vs CHL: 2.2±0.2, t(22) = 0.3, p = .79; Fig. 3B (*x-axis*)). Neither the proxy for performance during training (Fig. 3A), nor the proxy for proficiency during initial testing (Fig. 3B) were correlated with best detection thresholds for either group (Training CTR: R^2^ = 0.004 p = .86, CHL: R^2^ = 0.001 p = .93; Initial Testing CTR: R^2^ = 0.05 p = .49, CHL: R^2^ = 0.008 p = .79). This suggests that the higher 5 Hz detection thresholds in CHL animals could not be attributed to differences in training or the ability to perform the task.

It is possible that the sAM detection threshold differences were related to a decline in attention to the task. In behavioral studies, a common proxy for attention is the false alarm rate (in this study, the rate at which animals broke contact with the spout during safe trials). Figure 3C shows that the average false alarms across sAM depths over all days was quite low and, more importantly, did *not* differ between groups (CTR: 0.075±0.009 vs CHL: 0.090±0.012, t(22) = 1.0, p = .32). The same was true for average false alarms over the last three days of performance (CTR: 0.070±0.011 vs CHL: 0.098±0.014, t(22) = 1.6, p = .12).

A decline in general motivation could also have contributed to poorer performance by CHL animals. To assess this, we asked whether CHL animals performed fewer trials per session, as compared to CTRs. Figure 3D indicates that this was not the case: there was no difference in the number of trials performed by the two groups averaged over all days of repeated testing (CTR: 33.8±2.0 vs CHL: 35.8±3.1, t(22) = 0.54, p = .60). Additionally there was no relationship between number of trials performed and best detection threshold for either group (CTR: R^2^ = 0.12 p = .27, CHL: R^2^ = 0.04 p = .53).

### Auditory Cortical Responses to Static Tones in Awake CTR and CHL Animals

The behavioral findings suggest that early-onset bilateral auditory deprivation leads to a perceptual deficit in slow amplitude modulation detection. Since *in vitro* slice studies demonstrate that this form of moderate hearing loss affects synaptic function in cortex (e.g. [10]), we examined the effects on single ACx neuron response properties and asked whether these properties were correlated with behavior. However, the measures shown here do not, in themselves, imply that the ACx is the only locus of change.

**Figure 4:**
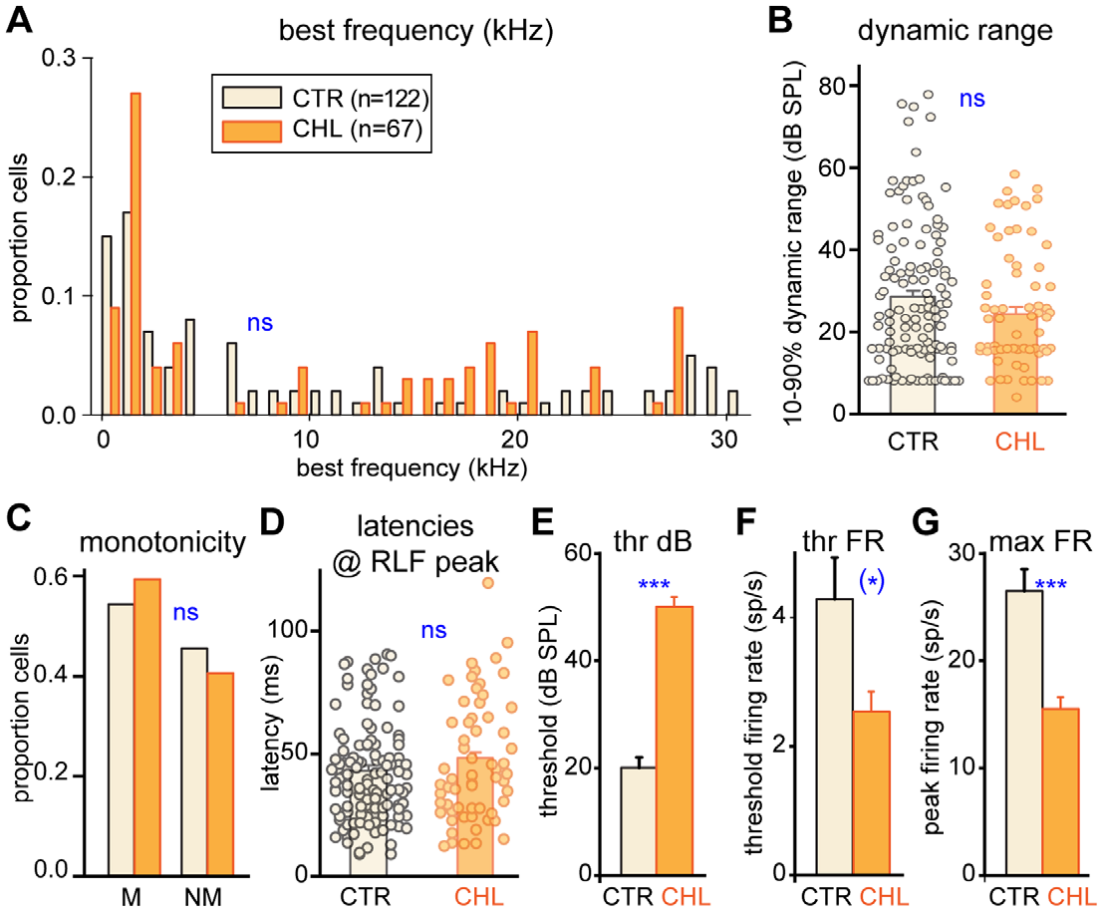
Limited effects of CHL on cortical responses to static tones. Cells were analyzed irrespective of responses to sAM, thus include a range of BMFs. (A) Cells were sampled across an equivalent frequency range, indicated by similar distributions of BF across groups. Rate-level functions were taken at each neuron’s BF. There were no group differences in (B) dynamic range, (C) the proportion of monotonic versus non-monotonic cells, or (D) the first-spike latencies when cells were driven most strongly, measured at the RLF peak. (E) As expected in the CHL animals, the level threshold across cells was higher than in CTRs. Group differences emerged based on firing rate: both (F) the firing rate at RLF threshold and (G) the maximal firing rate of the RLF were lower in CHL animals. *Black*  =  CTR; *Orange*  =  CHL. ***  =  p<.001; (*)  =  p = .05.

Single-unit recordings were obtained from primary ACx of awake untrained adult animals with treatments that paralleled the behavioral groups: 15 control animals and 8 animals with bilateral CHL induced at P10. The control cells include those presented in a previous study along with additional control neurons. The best frequency (BF) was determined manually, and rate-level functions (RLFs) were collected using 200 ms tone pips at BF for 122 CTR and 67 CHL neurons. The distribution of BFs did not differ between groups (Fig. 4A; t(187) = 0.37, p = 0.71), indicating that we sampled from a similar distribution of cells across the ACx tonotopic axis. Hearing loss did not affect cortical neurons’ dynamic range (Fig. 4B; t(187) = 1.0, p = 0.3) or the ratio of monotonic to non-monotonic cells (Fig. 4C; χ^2^ = 0.43, p = 0.51).

The first spike latency to tone pips that elicited the most spikes (i.e., pips at the peak of the RLF) did not differ across groups (Fig. 4D: CTR: 43.1±2.0 ms vs CHL: 47.4±3.2 ms, t(187) = 1.2 p = .23). As expected, absolute thresholds were significantly higher in CHL neurons (Fig. 4E; CTR: 20.1±1.9 dB SPL vs CHL: 49.1±1.7 dB SPL, t(187) = 9.8 p<.0001). Finally, tone pips evoked higher firing rates in CTR versus CHL neurons; this was marginally significant at threshold levels (Fig. 4F; t(187) = 1.9 p = .057) and significant at peak levels of the RLF (Fig. 4G; t(187) = 3.8 p = .0002).

### Developmental Hearing Loss-induced Changes in Cortical sAM Coding

The behavioral measures (Fig. 1) indicate that, for animals with developmental CHL, sAM detection is poorer at a slow modulation frequency (5 Hz), but is unaltered at a faster MF (100 Hz). We examined whether behavioral sensitivity levels were reflected in the sensitivity of ACx neurons. We recorded from 64 CTR and 53 CHL ACx neurons in untrained awake gerbils, and obtained responses to sAM tones at each neuron’s BF, presenting MFs ranging from 1 to 100 Hz to obtain a modulation transfer function (MTF). Responses were quantified based on firing rate, vector strength, and power at the sAM modulation frequency (see Methods). Firing rate ignores temporal properties, while vector strength measures phase-locking to the MF (e.g., high values signify discharge at a particular phase of the modulation cycle). Power provides the magnitude of the spiking response at the MF independent of phase. Thus, power incorporates both firing rate (response magnitude) and firing pattern information, and is well suited to quantify the neural responses that reflect the shape of the sAM envelope.

**Figure 5:**
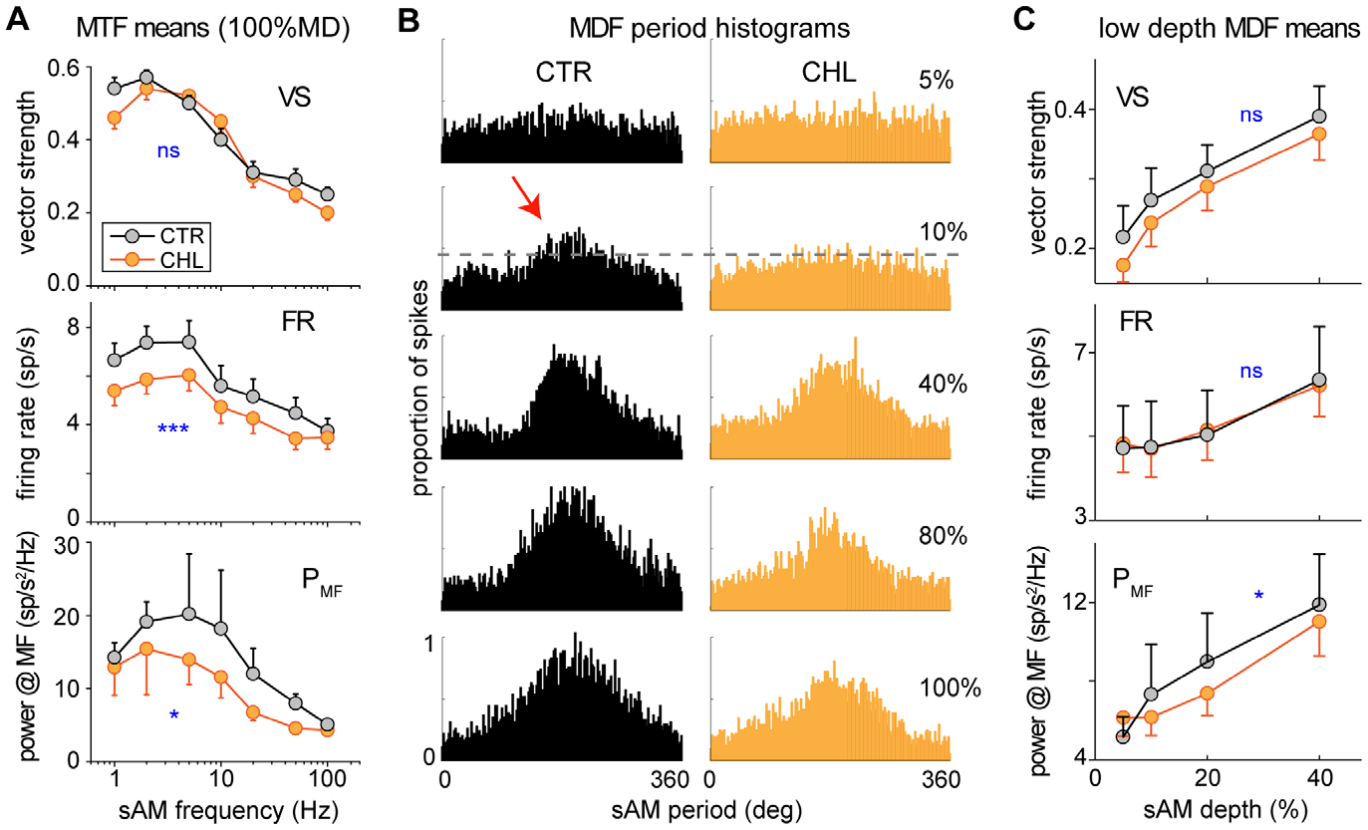
CHL alters cortical responses to sAM. (A) Modulation transfer functions (measuring responses across modulation frequency) differed across CTRs and CHLs for response measures that include strength of firing: firing rate (middle) and power at the stimulus MF (bottom). They did not differ for the response measure of synchrony: vector strength (*top*). Unlike at slow MFs, at 100 Hz sAM the response measures overlapped across the groups. (B) Population period histograms for those cells with a BMF of 5 Hz. Histograms were normalized for number of cells in each group, and are shown for several sAM modulation depths. The envelope shape of the sAM is increasingly apparent in the histograms at larger depths, for both groups. Qualitatively, in the CTR population a visible envelope shape emerges at a lower depth (10%), and the magnitude of the envelope is larger at higher depths. (C) Neural responses over the range of depths where behavioral thresholds differ were stronger for CTRs when measured by power (*bottom*), the measure that best reflects envelope shape. *Black*  =  CTR; *Orange*  =  CHL. ***  =  p<.001; *  =  p≤.05.

Figure 5A depicts MTFs using the mean values across cells for each of the three measures. A similar form was apparent for each measure: cortical responses to sAM have a low- or band-pass form. When comparing CTR and CHL responses, vector strength displayed equivalent phase-locking across all MFs (Fig. 5A *top panel*; F(1,1) = 0.83 p = .36). In contrast, firing rate and power measures showed stronger responses in CTR cells than in CHL cells (FR: F(1,1) = 8.9 p = .003, P_MF_: F(1,1) = 2.9 p = .05). Consistent with behavioral observations (Fig. 1), CHL responses were less robust at slower MFs, but equivalent to CTR responses at faster MFs (Fig. 5A *bottom two panels*) for measures that include response magnitude but not pure phase-locking.

To assess whether CHL reduced modulation depth sensitivity in ACx neurons, we identified the subset of ACx neurons with a best MF of 5 Hz (27 CTR and 36 CHL), and obtained responses to sAM tones at each neuron’s BF. The population response was quantified and compared for the two groups. Modulation period histograms were generated for each modulation depth, then summed across cells and normalized by cell number to create pooled MPHs for both groups (Figure 5B). A periodic shape was apparent in the temporal pattern of discharge of each population histogram, and the magnitude of the response increased with MD. At a qualitative level, a visible envelope response emerged at a shallower depth for the population of CTR cells vs CHL cells (Fig. 5B, *arrow*). A group difference in response magnitude across the lower range of depths is most relevant to the behavioral difference in detection thresholds (Fig. 1C). The mean values for all three measures across MDs ranging from 5%–40% are shown in Figure 5C. The hearing loss group responses over this range of depths were less sensitive than those of controls when measured by power, the measure which best represents the envelope response shape (VS: F(1,1) = 1.5 p = .21, FR: F(1,1) = 0.7 p = .39, P_MF_: F(1,1) = 4.3 p = .03). In general, cortical responses to sAM mimic the shape of the signal envelope, although there are a wide variety of periodic discharge patterns [36], [46]. The power measure is relatively robust to this response diversity. Therefore, we chose to use power when comparing behavioral and neural detection sensitivity across separate animals.

### Psychometric and Neurometric Measures Correlate with Hearing Status

A major goal of the study was to compare neural and behavioral sensitivity. Therefore we transformed the 5 Hz sAM-evoked spike trains into d′ curves for individual neurons, a form that could be compared directly to the behavioral data. The response for each MD was compared to the control response, i.e., to the smallest MD presented (5% MD). We defined d′ = 1 as threshold for detection of the signal to match the behavioral definition of threshold.

**Figure 6:**
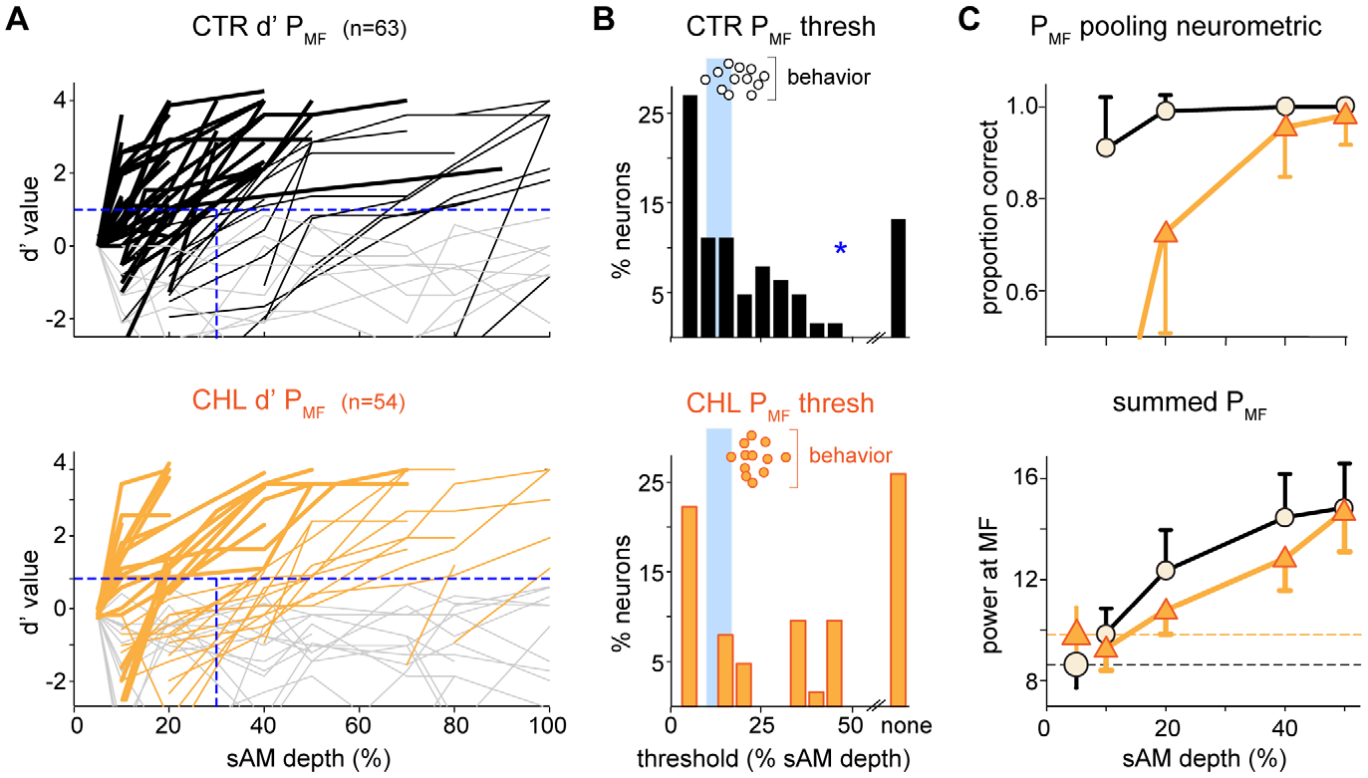
Neurometrics and psychometrics correlate with CHL treatment. Cells with BMFs of 5 Hz are compared with 5 Hz behavioral detection. (A) Individual neural d′ curves calculated from power at the modulation frequency (P_MF_) were truncated by displaying only that portion that crossed threshold (*horizontal dashed line*) until the peak d′ value was attained, with all curves truncated at d′ = 4.0. Neurons whose curves never reached threshold are in *grey*. In CTRs (*top, black*) there are visibly more cells with sensitive thresholds than in CHL animals (*bottom, orange*): cells with thresholds <30% MD (*vertical dashed line*) are shown with thick lines. (B) The distribution of P_MF_-based neural d′ thresholds (*bars*) was shifted significantly toward lower modulation depths for CTRs compared with CHLs. The behavioral performance of CTR and CHL animals (*circles*) is plotted at the top of each graph. The *blue shaded bars* indicate the region of CTR thresholds that do not overlap those of CHLs. (C) A pooling neurometric that represents a downstream neuron’s input summed across cells reveals better detection thresholds at low modulation depths for CTR versus CHL animals (*top*). The summed activity from which detection is computed is shown in the *bottom* graph. The larger symbols and dotted lines represent the baseline power in response to the lowest tested modulation depth, against which the other values were compared. *  =  p<.05.

Individual d′ curves based on the power measure are shown in Figure 6A. To focus on threshold performance, each curve displays only the portion that crossed threshold (*horizontal dashed line*) and is truncated at the peak d′ value attained, up to a maximum of d′ = 4. Neurons which never reached threshold at any MD are shown in *gray*. Fewer neurons with very sensitive thresholds (<30%, *vertical dashed line*) were observed in CHL animals (35%) compared with CTRs (52%). Figure 6B shows that the distribution of power-based d′ thresholds is significantly higher for CHL animals (t(115) = 2.1 p = .04). The practiced behavioral thresholds for CTR and CHL groups from Figure 1 are replotted in Figure 6B over the neural thresholds. The *vertical shaded bars* show the region of CTR thresholds that does not overlap with CHL thresholds. While there was a large overlap, individual values extended to lower values for CTR animals and to higher values for CHLs. Behaviorally, practiced CTR and CHL animals differ in performance by ∼4% MD (measured either as best day or mean of last 3 days performance). The magnitude of this behavioral difference is reflected in neurons that crossed threshold, as the average neural threshold across groups differs by ∼3% (CTR 30.9±3.2% MD vs CHL: 33.8±3.8% MD). Notably, none of the animals achieved thresholds as low as those of the best neurons, implying that neither group takes full advantage of the most sensitive cortical cells.

The neural d′ is a measure of within-cell sensitivity, which assumes that individual neurons are capable of signal detection. An alternative neurometric relies on the convergent neural architecture of a downstream neuron that sums across a pool of cells, “detecting” a signal when the summed response is larger than that elicited by the background signal (e.g., an unmodulated sound). This convergent architecture does not require individual cells to be good detectors, as detection can emerge across a population of cells. We tested whether this architecture would predict hearing loss-induced differences in sAM detection by applying this pooling neurometric to cortical responses based on the power measure. We chose to use power because this measure includes both firing rate and periodicity information and therefore represents the envelope shape of the response. This is particularly important for lower MFs where cortical neuron discharge rate co-varies with AM stimuli with idiosyncratic phase-specific responses [36], [46]. Also, the depth function based on power revealed a CHL-induced neural difference (Fig. 5C). For each MD, the power response was summed across all cells, and compared with the summed response magnitude to background (5% MD), where a higher sum was considered a correct detection. Figure 6C *top* illustrates the summed values from each depth that are compared with the lowest depth: each depth must be only just larger than baseline (represented by the *dashed lines*) to yield correct detection. The summed functions approximate the mean power functions in Figure 5C. A gradual increase in power is converted by pooling into a sharp rise in detection performance (Fig. 6C *bottom*). At lower depths, CHL cells did not distinguish sAM from background as well as CTR cells. Thus whether assessing the distribution of d′ thresholds or a pooled neurometric, there is less information available in cortex to animals reared with hearing loss.

## Discussion

Developmental auditory deprivation induces anatomical and physiological effects that persist in adult animals, suggesting that perception should also be affected. To test this idea, we examined the correlation between behavioral and neural measures of sAM detection in animals reared with CHL. When tested with a conditioned avoidance procedure, adult animals reared with CHL displayed significantly poorer detection thresholds for a slow (5 Hz) modulation, as compared with CTR animals. However, detection thresholds for a fast (100 Hz) modulation were equivalent (Fig. 1). This performance difference could not be attributed to overall differences in hearing level, task performance at the largest depths, strategy, or proxies for attention and motivation (Figs. 2, 3). Since our testing regimen challenges animals with increasingly difficult AM depths on successive days, we can state that asymptotic performance is better in control versus CHL animals. However, this outcome could result either from a stable perceptual impairment, or limitation in the amount of improvement that can be achieved through practice (i.e., perceptual learning).

Consistent with the diminished behavioral performance in CHL animals, single auditory cortex neuron responses to slow, but not fast, modulated sAM tones were significantly poorer in CHL animals when evaluated with metrics that incorporated firing magnitude (Fig. 5A). Compared with CTRs, the d′ neurometric revealed significantly fewer CHL neurons with low sAM detection thresholds (Fig. 6A,B). Similarly, a pooling neurometric revealed poorer detection performance across the population of CHL neurons (Fig. 6C). Cortical responses to unmodulated tones were consistent with this finding: CHL neurons displayed significantly reduced firing rates at equivalent sensation levels, as compared to CTRs; however, other response metrics did not differ between groups (Fig. 4). Taken together, the hearing loss-induced behavioral impairment in temporal detection can be attributed, in part, to a decline in sensory information about sAM depth. The impairments reported here were produced by a CHL induced during early postnatal development. The question remains whether similar changes might be produced by a CHL incurred during some later stage of development or in adulthood. Since our primary intent was examine the relationship between neural and behavioral metrics in a manipulated system, future studies will address variables such as age of hearing loss onset, duration of hearing loss, and age at which function is restored.

### Binaural Deprivation Effects on Neural Coding and Perception

Conductive hearing loss is associated with anatomical and physiological changes, from spiral ganglion cells to the auditory cortex [47], [48], [49], [50]. However, there are few studies that relate neural outcome measures to behavior. Unilateral deprivation is closely associated with deficits in sound localization and unmasking tasks that involve binaural integration [4], [6]. One experiment examined the behavioral effects of binaural deprivation with ear plugs, resulting in impaired frequency discrimination that was not attributable to a cochlear deficit [51]. Therefore, our findings that CHL-induced effects were similar across behavioral and physiological measures are broadly consistent with the literature. CHL reduced behavioral detection d′ thresholds by ∼4% MD, and physiological d′ thresholds by ∼3% MD. Although we do not know whether the perturbed coding properties recorded in ACx are the exclusive source of behavioral differences, they are a plausible source, as temporal processing is reliant on auditory cortical activity [52], [53], [54], [55].

The rationale for recording neural responses in ACx is that it reflects both information generated locally and as well as that inherited from subthreshold inputs. Therefore, recording from sensory cortex allows us to assess the information available to higher decision-making and pre-motor areas [56], [57]. Differences in the AM-related information available in ACx is likely to reflect that available in the entire system. A second reason for recording in ACx is that CHL, identical to that studied here, produces synaptic and biophysical changes in ACx [10], [12], [13], and at least some of these persist in adults [14]. Thus ACx should be one neural region that would explain differences in encoding and ultimately perception. Furthermore, synaptic changes in ACx were not induced by adult-onset CHL [58]. If these and similar changes subserve the effects seen here, adult-manipulated animals would not be expected to differ from controls. Testing adult-onset CHL animals will establish whether hearing loss alone is responsible for the behavioral and neural deficits, or whether the manipulation must occur during development. Ultimately, our data indicate only that auditory deprivation diminishes the neural information about slow sAM signals that is available to the animal.

Our physiological recordings were obtained from awake untrained animals, and our neural data indicated that CHL animals have diminished sensory information available to them prior to any training (Fig. 6). It remains to be tested whether training would increase or decrease the magnitude of this deficit. Training can rescue performance and alter tonotopicity and receptor expression in noise-reared animals [59], [60], [61]. However, it is unknown whether training on an auditory detection task would have the same effect on the neural properties of animals that were manipulated during development, as it would on normal animals. To this point, functional assessments in deprived animals have only employed anesthetized, untrained animals. Although more challenging, the ideal comparison to behavior would be to record from performing, rather than passively listening, trained animals, since responses depend on behavioral state [62], [63]. Experiments in awake, performing animals can clarify whether neural encoding continues to reflect behavioral deficits following a period of training.

The auditory stimuli used for behavior differed from those used for physiology: for behavior, sAM noise was preceded by broadband noise, whereas for physiology, sAM tones at BF were preceded by silence. A noise carrier was used for the behavior to eliminate the potential for sidebands to evoke a pitch percept at higher MFs, and force the animals to rely on temporal cues. Therefore, it is important to consider whether the differences observed for neural sensitivity could have been enhanced or diminished by the choice of stimulus parameters. The first concern is that the behavioral stimuli contained no sudden transient increase in stimulus energy. To minimize the contribution of the transient onset component to our neurophysiological results, we excluded the first period of each sAM response from our analyses. A second concern is the relative level of response adaptation that would be induced by a continuous signal (behavior) versus a transient signal (neurophysiology). In fact, in vitro analyses suggest that short tem depression of cortical synapses is greater following hearing loss [10], [12]. Thus, had we used continuous stimuli for the neurophysiological assay, we might have expected a greater difference between control and CHL neurons; that is, we may have underestimated the neural coding changes effected by CHL. A third concern is the difference in carrier. Published results suggest that there is considerable between-neuron response variability whether AM responses are assessed with tonal or noise carriers. In separate studies, AM responses were characterized in the same, awake species (Rhesus macaque), using tone or noise carriers; in a different study, noise and tone carriers were compared in the same anesthetized species (cat) [64], [65], [66]. There were no systematic differences in responses to noise- versus tone-carrier sAM that would lead us to expect a different result had we chosen to use a noise carrier for our neurophysiological assay. Therefore, we conclude that the differences in neural coding reflect the effects of prolonged hearing loss, although there is some reason to believe that we have underestimated the magnitude of the effect.

### Effect of Hearing Loss on Modulation Frequency Threshold

Amplitude modulation detection thresholds are consistently higher for faster modulation frequencies across many species. Thresholds for 100 Hz MF are typically 5–15% MD higher than those for 5 Hz MF [21], [29], [67]. This was the case for CTRs, but did not hold true for CHL animals. CTR thresholds were significantly lower (by ∼9% MD) for 5 Hz than for 100 Hz, but CHL thresholds were not different across these two modulation frequencies (Fig. 1). Thus, CHL animals were as good as CTRs in detecting fast modulations, and impaired only for slow modulations. This poor performance for the CHLs at the lower MF suggests that early-onset hearing loss changes the shape of the temporal modulation transfer function, rather than shifting the entire curve towards higher thresholds. Cortical MTFs parallel this result: while both CTR and CHL MTFs showed bandpass characteristics typical of responses in many species, CHL responses were reduced at lower MFs more than higher (Fig. 5A). Notably, this behavioral effect has also been demonstrated in a study comparing hearing-impaired cochlear implant users: those with early hearing loss displayed higher sAM thresholds primarily at slower modulation frequencies [25]. sAM detection in adult humans with sensorineural hearing loss (SNHL) is not significantly impaired, when effective bandwidth is controlled for, consistent with the idea that the developmental deprivation is necessary for an impairment [68], [69], [70]. Dyslexic children and adults are similarly impaired in detecting slow compared with fast modulations [71], [72], [73], [74]. One implication of this result is that low MF detection is a separate neural process from high MF detection, and that the development of this network is more vulnerable to disuse or other disorders.

### Locus of Neural Impairment

In principle, the poorer sAM detection thresholds displayed by ACx neurons could be due to changes anywhere along the auditory pathway. To address this issue, a comparison can be made to recordings from auditory nerve (AN) fibers. Although AN responses to sAM stimuli following developmental CHL are not available, there is a quantitative assessment of sAM coding in adult animals with noise-induced SNHL. For AN fibers with increased thresholds, envelope coding at 100% MD is enhanced (i.e., better phase-locking) compared to control AN fibers [75]. The AN response characteristics following SNHL differ from those reported here for developmental CHL. They are more consistent with the lack of sAM impairment in SNHL adults, particularly with one study showing increased sAM sensitivity at very low stimulus amplitudes [69]. Although this may reflect a differential effect of age of hearing loss, there are other fundamental dissimilarities in experimental design (e.g., the AN recordings presented 100% MD sAM to anesthetized chinchillas with non-developmental noise-induced hearing loss).

The effects reported here may be due, in part, to changes in central processing. The CHL surgery (malleus removal) attenuates sound transmission to the inner ear, but should not compromise the cochlea: ossicle removal does not change hair cell counts on the basilar membrane, and bone conduction thresholds are reported to be normal [76]. At the same time, it remains possible that unintended outer hair cell damage could have affected the encoding of variations in sound amplitude via reduction of cochlear compressive non-linearities [27]. However, the overall hearing sensitivity of CHL animals was not correlated with their sAM detection thresholds, as measured by either ABRs or behavioral hearing thresholds (Fig. 2). Finally, developmental CHL affects EPSP amplitudes and short-term synaptic depression in pyramidal neurons, and prevents the maturation of inhibitory interneurons [10], [12], [14], suggesting that changes to the cortical network could contribute to the reduced firing rate and poorer sAM envelope encoding. The reduced overall firing rates in CHL animals may be attributable to a reduction in sound-evoked drive via greater short-term depression of thalamic excitatory afferents to the ACx [10] and/or lemniscal excitatory afferents to the IC [77]. It appears that the loss of inhibitory strength, both at the level of the IC and ACx [58], [78] is not sufficient to restore a normal discharge rate, at least for AM stimuli.

### Perceptual Processing with Hearing Loss: Sensory Versus Non-sensory Factors

Our interpretation of the results is that developmental hearing loss leads to a sensory processing deficit that can explain poorer performance on perceptual tasks. However, the results do not address alternative interpretations that involve non-sensory factors. For example, hearing-impaired listeners expend extra listening effort to achieve speech comprehension equivalent to that of normal listeners, as revealed by poorer performance on secondary cognitive tasks [79], [80], [81], [82], [83], [84]. Differences in auditory-based learning, attention, or memory mechanisms may also play a role [85], [86], [87]. For example, children with hearing loss can display poorer verbal short-term memory and phonological discrimination than controls [88], [89]. In this study, proxies for attention and motivation (Fig. 3) did not reveal a difference between CTR and CHL animals. Finally, it is possible that individuals with hearing loss may access their existing sensory information less effectively. Reduced speech intelligibility due to hearing loss or stimulus complexity can decrease activation in sensory cortices [90], [91], [92]. Thus, cognitive processes are likely modulating lower-level sensory regions during effortful processing to assist with perception. To tease out the relative contributions of sensory and cognitive factors to perception in animals reared with hearing loss, future experiments could obtain recordings from behaving animals while manipulating motivation or effort.
